# Increased maternal consumption of methionine as its hydroxyl analog improves placental angiogenesis and antioxidative capacity in sows

**DOI:** 10.1186/s40104-025-01159-z

**Published:** 2025-03-09

**Authors:** Rui Zhou, Shanshan Lai, Peiqiang Yuan, Li Zhe, Lunxiang Yang, Yves Mercier, Liang Hu, Xiaoling Zhang, Lun Hua, Yong Zhuo, Shengyu Xu, Yan Lin, Bin Feng, Lianqiang Che, De Wu, Zhengfeng Fang

**Affiliations:** 1 Key Laboratory for Animal Disease-Resistance Nutrition of China Ministry of Education, Animal Nutrition Institute, Sichuan Agricultural University, 211 Huimin Road, Wenjiang District, Chengdu, 611130 People’s Republic of China; 2 Key Laboratory of Agricultural Product Processing and Nutrition Health, Ministry of Agriculture and Rural Affairs, College of Food Science, Sichuan Agricultural University, Ya’an, 625014 People’s Republic of China; 3 Adisseo France S.A.S, CERN, Commentry, France

**Keywords:** Angiogenesis, Gestation sow, Hydroxy-methionine analogue, Placenta, TMT Proteomics

## Abstract

**Background:**

Previous evidence suggests that methionine (Met) consumption can promote placental angiogenesis and improve fetal survival. To investigate the mechanisms by which increased levels of Met as hydroxyl-Met (OHMet) improve placental function, forty sows were divided into four groups and fed either a control diet, or diets supplemented with 0.15% OHMet, 0.3% OHMet or 0.3% Met (*n* = 10). Placentas were collected immediately after expulsion, and extracted proteins were analyzed by tandem mass tag based quantitative proteomic analysis.

**Results:**

We found that 0.15% OHMet consumption significantly increased placental vascular density compared with the control. Proteomic analysis identified 5,136 proteins, 87 of these were differentially expressed (*P* < 0.05, |fold change| > 1.2). Enriched pathways in the Kyoto Encyclopedia of Genes and Genomes for 0.15% OHMet vs. control and 0.15% OHMet vs. 0.3% OHMet were glutathione metabolism; for 0.15% OHMet vs. 0.3% Met, they were NOD-like receptor signaling and apoptosis. Further analysis revealed that 0.15% OHMet supplementation upregulated the protein expression of glutathione-*S*-transferase (GSTT1) in placentas and trophoblast cells compared with the control and 0.3% OHMet groups, upregulated thioredoxin (TXN) in placentas and trophoblast cells compared with the 0.3% OHMet and 0.3% Met groups, and decreased reactive oxygen species (ROS) levels in trophoblast cells compared with other groups. In contrast, sows fed 0.3% OHMet or 0.3% Met diets increased placental interleukin 1β levels compared with the control, and upregulated the protein expression of complex I-B9 (NDUFA3) compared with the 0.15% OHMet group. Furthermore, homocysteine, an intermediate in the trans-sulphuration pathway of Met, damaged placental function by inhibiting the protein expression of TXN, leading to apoptosis and ROS production.

**Conclusion:**

Although dietary 0.15% OHMet supplementation improved placental angiogenesis and increased antioxidative capacity, 0.3% OHMet or 0.3% Met supplementation impaired placental function by aggravating inflammation and oxidative stress, which is associated with cumulative homocysteine levels.

**Supplementary Information:**

The online version contains supplementary material available at 10.1186/s40104-025-01159-z.

## Introduction

The placenta plays a crucial role in nourishing the fetus and promoting its growth [1, 2]. Placental activities directly affect the well-being of the fetus [3]. Accumulating evidence indicates that the physiological mechanisms governing placental formation, growth, and development are intricately linked to specific factors such as angiogenesis and antioxidant abilities [4]. These factors significantly influence pregnancy outcomes. Amino acids play a vital role in promoting placental growth by facilitating protein synthesis and supporting trophoblast and endothelial cell proliferation [5]. Growing evidence suggests that methionine (Met), an essential amino acid, improve placental angiogenesis and antioxidant defense [6]. Previous studies showed that the Met intake recommended by the National Research Council (2012) [7] for gestating sows was insufficient for optimal reproductive performance [8, 9]. However, our previous study and others found a progressive increase in maternal homocysteine (Hcy) levels during late gestation [10, 11]. Moreover, elevated dietary Met concentration could lead to Hcy accumulation, thereby disrupting the placental function [12]. Therefore, preventing Hcy accumulation while ensuring Met supply is essential for placental angiogenesis and other functions.

DL-2-hydroxy-4-methylthiobutanoic acid (OHMet), a new source of Met, is widely used in commercial animal feeds [13–15]. Notably, dietary OHMet supplementation accumulates lower Hcy levels when compared to crystalline D-Met, reflecting the metabolic difference between the two sources of Met [13, 16]. Moreover, Met is utilized more in the intestine than OHMet, so Met has a higher first-pass metabolic rate [16]. Our recent studies also demonstrated that dietary 1.5 g/kg OHMet supplementation improved maternal metabolism and increased the fetal survival rate [17]. However, the mechanisms through which different Met sources and levels influence placental function are not fully understood.

Therefore, this study aimed to explore the potential for enhancing placental morphology and function through increased maternal OHMet intake. Placental analysis in sows was performed using tandem mass tag (TMT) based quantitative proteomics, along with assessments of antioxidant capacity, morphology, and gene expression associated with antioxidant and angiogenic functions in porcine trophoblast (pTr) cells. These findings may enhance our understanding of the biological mechanisms that underlie the placental function of maternal Met nutrition.

## Materials and methods

The experimental scheme was approved by the Institutional Animal Care and Use Committee of Sichuan Agricultural University (Ya’an, China) (approval No. SYXK-Chuan-2014-184). OHMet was provided by Adisseo (88%, Rhodimet AT88, Adisseo Life Science, Shanghai, China) and the L-Met (99%) was obtained from Cheil Jedang Biochemical Co., Ltd. (Qingdao, China).

### Animals, experimental design and diets

Forty primiparous sows [Duroc × (Landrace × Yorkshire)] with similar BW (154.46 ± 1.60 kg, *P* = 0.85) were selected and randomly assigned into 4 treatments, each with 10 replicates, starting on d 1 of gestation (G1). The average litter size of the sows was 12.13 ± 0.50. All sows were fed an equal amount control (CON) diet until G59. The CON diet was formulated to meet the nutrition requirements of gestating sows, as recommended by the NRC (2012) [7]. The present study was divided into 2 phases: the first phase spanned from G60 to G90, and the second phase from G90 to G114. Sows in the CON group were fed a basal diet containing 3.4 g of Met per kg of feed during phase 1 and 4.5 g during phase 2 (Table 1). The other groups were: the 1.5S-OHMet group, supplemented with 1.5 g OHMet (88% Purity) per kg of feed; the 3.0S-OHMet group, supplemented with 3.0 g OHMet per kg of feed; and the 3.0S-Met group, supplemented with 3.0 g L-Met (99% Purity) per kg of feed.

**Table 1:** Ingredients and nutrient composition of the experimental diets (as-fed basis)

Feedstuff, g/kg	Phase 1	Phase 2	Nutrient level^a^	Phase 1	Phase 2
	G60–G90	G91–G114		G60–G90	G91–G114
Corn	689.11	642.58	Gross energy, MJ/kg	16.51	16.52
Wheat bran	70.00	55.00	Crude protein, %	10.13	13.43
Rice bran meal	60.00	60.00	SID-Lys, %	0.52	0.69
Soybean meal	41.50	97.00	SID-Met, %	0.15	0.20
Bean curd	69.30	50.90	SID Met + Cys, %	0.34	0.45
Fish meal		20.00	SID-Thr, %	0.37	0.48
Soybean oil	40.00	40.00	SID-Trp, %	0.09	0.13
L-Lys-HCl (98.5%)	2.602	2.324	SID-Ile, %	0.30	0.36
L-Met (99%)	0.112	0.469	SID-Val, %	0.37	0.49
L-Thr (98.5%)	0.892	0.966	Calcium, %	0.61	0.83
Trp	0.084	0.15	Total phosphorus, %	0.49	0.62
Dicalcium phosphate	8.041	10.528	STTD-P, %	0.27	0.36
Limestone	8.733	10.461			
Sodium chloride	4.00	4.00			
Choline chloride (50%)	1.70	1.70			
Premix^b^	3.925	3.925			
Total	1,000	1,000			

Gx = day x of gestation

^a^Gross energy is analyzed value and the others are calculated values

^b^Vitamin and mineral mixture for gestation sows supplied the following amounts of vitamins per kg and minerals per kg of complete diet: 6,000 IU vitamin A; 1,200 IU vitamin D_3_; 50 IU vitamin E; 1 mg vitamin B_1_; 3.6 mg vitamin B_2_; 1.8 mg vitamin B_6_; 12.5 μg vitamin B_12_; 20 mg niacin; 2 mg folate; 316 mg iron; 183 mg copper; 289 mg zinc; 169 mg manganese; 0.3 mg iodine

### Feeding management

Semen was obtained from three adult male pigs (Yorkshire) with of the same batch and age. The vitality of sperm was at least 85%, and the abnormality rate of the sperm did not exceed 10%. Sows were individually housed in pens measuring 2.6 m × 0.6 m from G1 to G107, and then moved to adjustable 2.2 m × 1.5 m farrowing cages. Each sow was housed in a single barn with controlled environmental conditions. Temperatures was maintained between 20–24 °C and humidity levels between 40%–60%. At parturition, the total number of litters, liveborn, mummies, and weak piglets were recorded, and piglet birth weight was measured after farrowing. Two sows were removed from the dataset due to death during farrowing.

### Data collection and sampling

Six to eight sows per group were randomly selected for sample collection. Fasting blood samples were collected from sows through the ear vein on the farrowing day. Blood samples were centrifuged at 3,000 × *g* for 15 min, after which the serum was then separated and stored at −20 °C until analysis, or at −80 °C for cell culture purposes. During delivery, the umbilical cord of each piglet was tied with labeled cotton thread to facilitate placenta matching. Placental samples were immediately collected from piglets whose BW was closest to the average, using the method outlined in previous studies [10, 11]. After the placenta was expelled, two sections of placental tissue were collected, each about 5 cm from the cord insertion point. One section was immediately frozen in liquid nitrogen, and the other was preserved in 4% paraformaldehyde.

### Determination of placental vascular density

Tissue samples were initially fixed with a 4% paraformaldehyde solution, embedded in paraffin, and then cut into 5 µm sections. The sections were subsequently stained with hematoxylin and eosin (H&E). Images of three to four fields on each slide with the stained tissues were captured using a Leica DM1000 projection microscope. The placental tissue area was delineated, and the placental vessels within this area were also delineated for the quantification of their area and number. Blood vessels within the stroma were imaged using a 4 × objective and 10 × eyepiece, while other vascular indicators were captured with a 40 × objective and 10 × eyepiece. Image analysis techniques (Image Pro Plus, Media Cybernetic, USA) were used to calculate the relative area of the placental stroma vessels. The width of the placental fold is the average distance from one fold to another. The length of the placental fold is the average distance from the top of all folds to the bottom [18]. The placental vascular density was defined as the area of the blood vessels divided by the area of the stroma, then multiplied by 100%.

### Measurement of antioxidative capacity and inflammatory cytokine in placenta

Placental tissue samples (approximately 0.1 g) were homogenized in an ice-cold 0.9% physiological saline solution (1:9, w/v), then centrifuged at 3,500 × *g* for 15 min at 4 °C. Placental samples were analyzed for glutathione peroxidase (GSH-Px), total superoxide dismutase (T-SOD), malondialdehyde (MDA), catalase (CAT) and induced nitric oxide synthase (iNOS) concentrations, following the method provided by Nanjing Jiancheng (Nanjing, China). Reduced (GSH) and oxidized (GSSG) glutathione were measured using a commercially available kit from Beyotime (Jiangsu, China). Placental tissue concentrations of tumor necrosis factor-α (TNF-α), interleukin-6 (IL-6), and interleukin-1β (IL-1β) were determined using commercial ELISA kits from Meimian (Jiangsu, China). The intra-assay coefficient of variation was less than 10%, and the inter-assay coefficient was less than 12%. Results were normalized against total protein concentration for comparisons between samples [19].

### *S*-adenosyl-methionine (SAM), *S*-adenosyl-homocysteine (SAH), and amino acid analysis

The SAM and SAH levels in serum samples were determined using ultra-performance liquid chromatography (UPLC, Waters, USA) equipped with a Waters ACQUITY UPLC BEHC18 column (150 mm × 2.1 mm, 1.7 μm). In brief, 400 μL of the serum sample and 40 μL trichloroacetic acid solution (400 g/L) were mixed and centrifuged at 14,000 × *g* for 20 min at 4 °C, and the supernatant was collected. The SAM (#A4377) and SAH (#A9384) standards were purchased from Sigma-Aldrich (Missouri, USA). Concurrently, serum amino acid concentrations were determined using an automatic amino acid analyzer (Hitachi L-8800, Hitachi High-Technologies Corporation, Tokyo, Japan) [17].

### Placental quantitative proteomics analysis

Samples from each group in the placenta, totaling three per group, were analyzed using the Tandem Mass Tags (TMT) method [20]. Total proteins were extracted from the samples. Following quality assessment (Fig. S1), the samples were labeled with TMT tags, desalted, and lyophilized. Fractions were separated, and liquid chromatography-tandem mass spectrometry (LC–MS/MS) was used to analyze the resulting peptides. After quality control, the protein data were searched against the Sus scrofa protein database using Proteome Discoverer 2.2 (Thermo Fisher Scientific). Protein expression levels were normalized relative to placental samples. The threshold of a fold change ≥ 1.20 (or ≤ 0.83) and a *P*-value ≤ 0.05 was used to identify differentially expressed proteins (DEPs). DEPs were identified across the four treatment groups. Functional enrichment analysis of the DEPs was conducted using the Gene Ontology (GO) database and the Kyoto Encyclopedia of Genes and Genomes (KEGG) database.

### In vitro cell model

The porcine trophoblast cell line (pTr) used in this experiment was previously established and characterized using porcine blastocysts collected on G12 [1]. The pTr cells were cultured in DMEM/F12 (Gibco, Missouri, USA) supplemented with 10% fetal bovine serum (FBS) (Gibco, Missouri, USA) and 1% penicillin–streptomycin liquid (Gibco, Missouri, USA). All maternal serum was filtered using a 0.22-μm sterile filter (Millipore, USA) before cell processing. For maternal serum treatment: pTr cells were seeded in 12-well plates and, after 24 h, the medium was switched from DMEM/F12 with 10% FBS to Met-free DMEM/F12 (Cellverse, Shanghai, China) supplemented with 20% maternal serum from each of the four groups [21]. For Hcy treatment, pTr cells were seeded in 12-well plates. After 24 h, the medium was changed from DMEM/F12 with 10% FBS to DMEM/F12 with varying Hcy concentrations. The cells were collected after 24 h of treatment for further analysis.

### Cell viability, proliferation and lactate dehydrogenase release assay

Cell viability was assessed using a CCK-8 kit (Beyotime) according to the manufacturer's protocols. Cell proliferation was determined by using a EdU-555 kit (Beyotime, #C0075S). Apoptosis-induced destruction of the cell membrane structure can result in the release of lactate dehydrogenase (LDH) from the cytoplasm. LDH activity was measured using an assay kit (Beyotime). Fluorescence was measured at 450 nm with a microplate reader. All procedures followed the manufacturer’s instructions.

### Intracellular ROS analysis and scratch healing assay

Intracellular levels of reactive oxygen species (ROS) were measured using DCFH-FA (Solarbio, Beijing, China) according to the manufacturer's protocols. pTr cells were seeded in a 6-well plate and cultured to form a confluent monolayer overnight. A scratch wound was then created with a 200-μL pipette tip, and the effect of Hcy on wound healing was measured 24 h post-scratch.

### Flow cytometry analysis of apoptosis

The fluorescence intensity of FITC Annexin V and propidium iodide (PI) was analyzed using a flow cytometer (BD, FACSVerse, USA). Apoptosis data were analyzed using FlowJo X software.

### Real-time quantitative PCR

Real-time quantitative PCR (RT-qPCR) was performed as follows. Total RNA was extracted from frozen placental samples using Trizol reagent (Sigma-Aldrich). Subsequently, cDNA was synthesized from 1 mg of total RNA using a PrimeScript reverse transcription kit (Takara, Dalian, China), following the manufacturer’s instructions. The cycling conditions were set as 95 °C for 15 s, followed by 40 cycles of 95 °C for 5 s, and then 60 °C for 34 s. Real-time PCR was conducted using a 7900HT ABI Prism system (Applied Biosystems, Foster City, CA, USA) and SYBR Green RT-qPCR reagent (Takara). β-Actin and GAPDH were used as internal reference genes. The RT-qPCR data were analyzed using the 2^−ΔΔCt^ method [22]. Gene expression in the placenta was assessed using an RNase protection assay. Primer sequences for the genes are listed in Table S1.

### Western blot analysis

Placental tissues and pTr cells were lysed using cold lysis buffer (Beyotime), centrifuged at 12,000 × *g* for 30 min at 4 °C, and the supernatant was combined with loading buffer (Bio-Rad, Hercules‌, USA) and mercaptoethanol, then boiled for 5 min. Samples were separated by SDS-PAGE using 10% or 15% acrylamide gels and transferred onto PVDF membranes. Primary antibodies were thioredoxin (TXN, Zen BioScience, 1:1,000), HADHA (Zen BioScience, 1:1,000), NDUFA3 (Zen BioScience, 1:1,000), ENO3 (Zen BioScience, 1:1,000), GSTT1 (Proteintech, 1:1,000), GAPDH (Absin, 1:1,000), β-actin (Cell Signaling Technology, 1:1,000). The secondary rabbit antibodies (1:2,000) were purchased from Beyotime. The protein bands were quantified by Image Lab (Bio-Rad).

### Statistical analysis

The data were analyzed using the PROC MIXED procedure of SAS 9.4 (SAS Inst. Inc., Cary, NC, USA). The Shapiro–Wilk test was conducted to assess the normality of the data. Multiple comparison of data among the groups were performed using Turkey’s tests when the data were normally distributed. Data points identified as outliers by the Grubbs test (α < 0.05) were excluded from the analysis. Proteomic data was analyzed using paired *t*-test. Pearson’s correlation analysis was used to identify relationships between differential proteins and placental traits, as well as between differential proteins and Met metabolites. Data are presented as means ± standard error of the mean (SEM). Statistical significance was defined as *P* < 0.05, while a tendency towards difference significance was indicated at 0.05 ≤ *P* < 0.10.

## Results

### Effects of increased consumption of Met as OHMet on placental angiogenesis

**Fig. 1:**
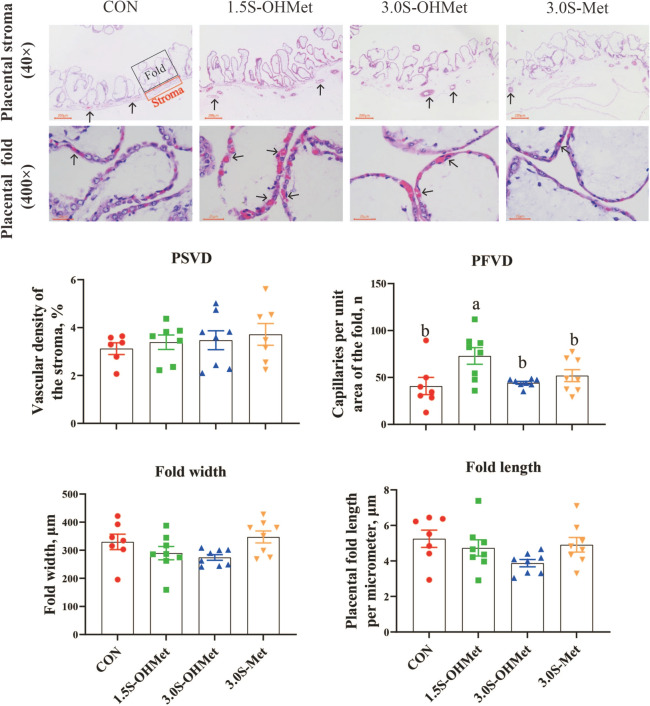
Effect of increased consumption of methionine as OHMet on placental angiogenesis. Data were presented as means with standard error of the mean (SEM). Abbreviations: CON = basal diet; 1.5S-OHMet = basal diet + 1.5 g/kg OHMet; 3.0S-OHMet = basal diet + 3.0 g/kg OHMet; 3.0S-Met = basal diet + 3.0 g/kg Met. PSVD = the vessel density of placental stroma (× 40 magnification; scale bar, 200 μm). PFVD = the capillaries per unit area of placental fold (× 400 magnification; scale bar, 50 μm). Significant difference among the four treatments is marked with different superscripts (*P* < 0.05) (*n* = 6–8)

To determine if increased maternal OHMet intake enhanced placental angiogenesis, placental vascular density was assessed. The findings indicated that dietary intake of 1.5S-OHMet elevated the number of capillaries per unit area in the placental fold compared to the CON, 3.0S-OHMet, and 3.0S-Met groups (*P* < 0.05, Fig. 1). However, no significant differences were found in the vascular density of the placental stroma, or fold width and length per micrometer of the placenta (*P* > 0.05, Fig. 1).

### Effects of increased consumption of Met as OHMet on placental anti-oxidative capacity and inflammatory cytokines

**Fig. 2:**
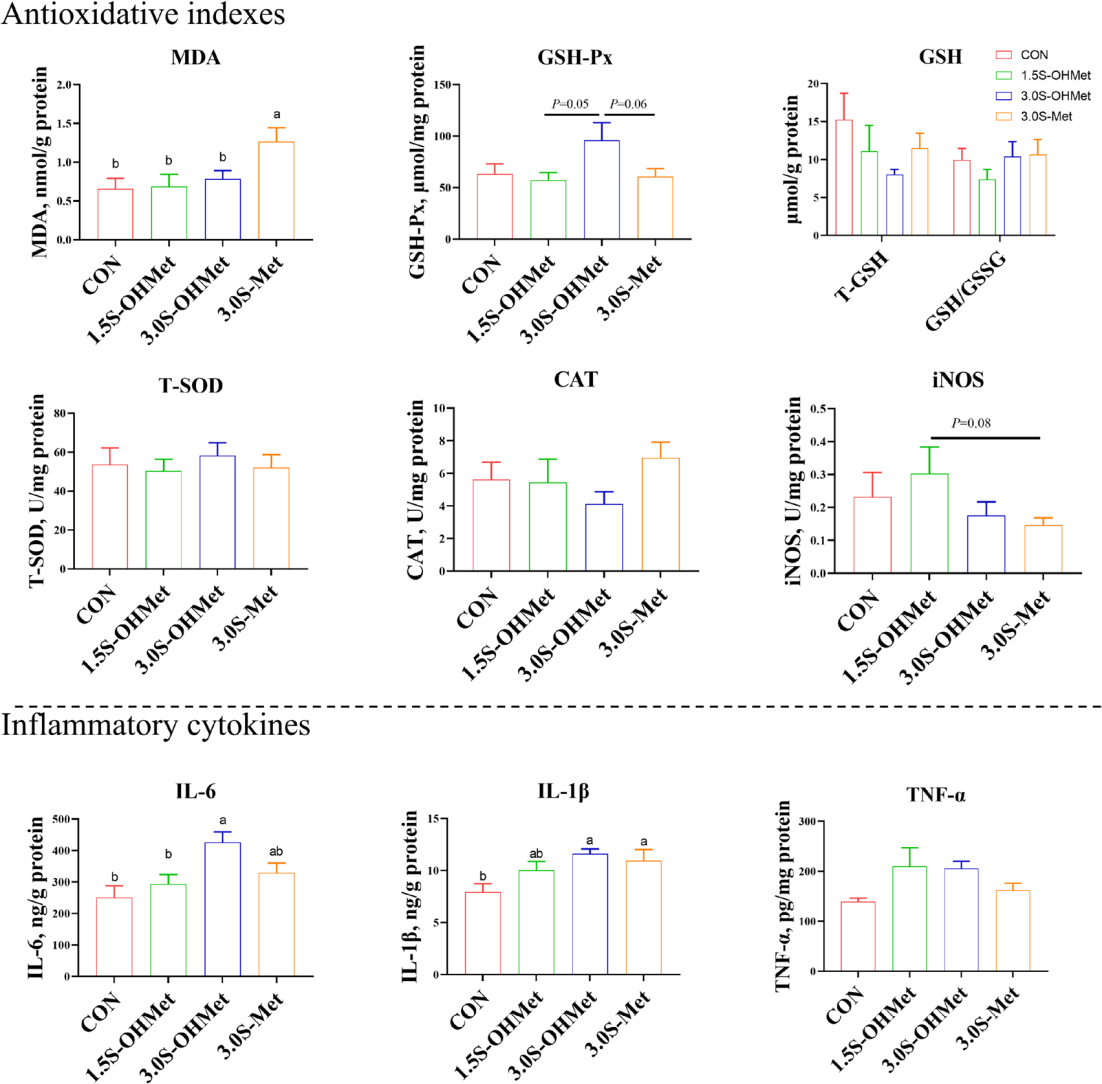
Effect of increased consumption of methionine as OHMet on placental antioxidative capacity and inflammatory cytokines. GSH-Px, glutathione peroxidase; SOD, superoxide dismutase; CAT, catalase; MDA, malondialdehyde; iNOS, induced nitric oxide synthase; GSH, reduced glutathione; GSSG, oxidative glutathione. IL-6, interleukin 6; IL-1β, interleukin 1β; TNF-α, tumor necrosis factor α. Data were presented as means with standard error of the mean (SEM). Significant difference among the four treatments is marked with different superscripts (*P* < 0.05) (*n* = 6–8). Abbreviations: CON = basal diet; 1.5S-OHMet = basal diet + 1.5 g/kg OHMet; 3.0S-OHMet = basal diet + 3.0 g/kg OHMet; 3.0S-Met = basal diet + 3.0 g/kg Met

To further investigate the impact of maternal Met supplementation on placental oxidative status, antioxidative parameters in the placenta were measured. As shown in Fig. 2, dietary 3.0S-Met consumption increased placental MDA levels (*P* < 0.05) compared to other groups and decreased placental GSH-Px activities (*P* = 0.05) when compared to the 3.0S-OHMet group. However, no significant differences were observed in the placental levels of T-SOD, CAT, iNOS, GSH and GSSG (*P* > 0.05). Furthermore, measurements of placental inflammatory cytokines revealed increased levels of IL-1β following consumption of 3.0S-OHMet or 3.0S-Met diets compared to the CON group (*P* < 0.05). Additionally, consumption of the 3.0S-OHMet diet increased placental IL-6 levels compared to both the CON and 1.5S-OHMet groups (*P* < 0.05). No differences were observed in placental TNF-α levels among the four treatment groups (*P* > 0.05).

### Placental proteomic analysis and identification of DEPs

**Fig. 3:**
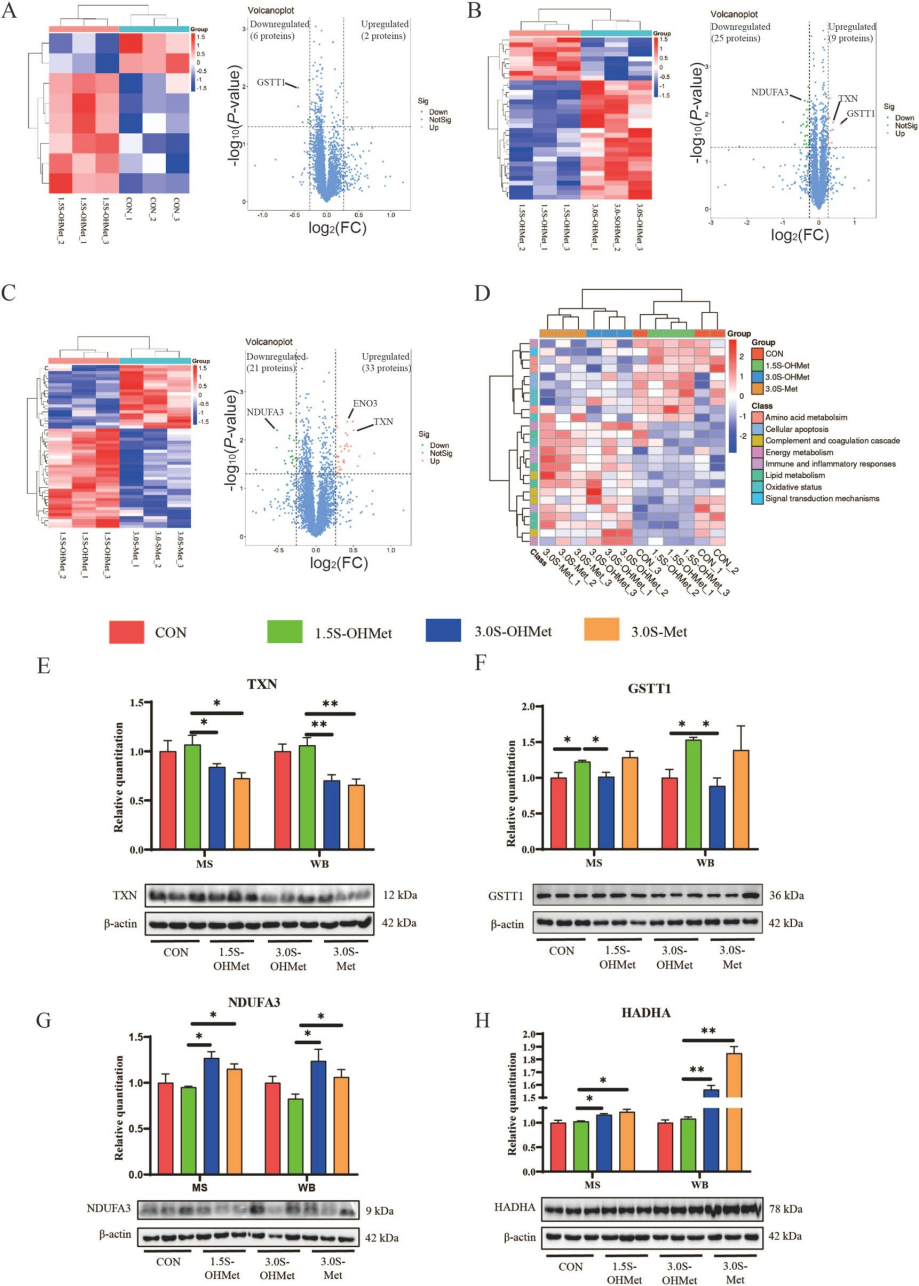
Effect of increased consumption of methionine as OHMet on placental proteins. **A**–**C** The cluster heat map and volcano map of differential proteins between CON and 1.5S-OHMet, 1.5S-OHMet and 3.0S-OHMet, and 1.5S-OHMet and 3.0S-Met. **D** Distribution of differential placental proteins in individual samples based on Pearson distance and average clustering. **E**–**H** Validation of four differentially expressed proteins from the results of mass spectrometry (MS) and western blotting (WB). TXN, thioredoxin; GSTT1, Glutathione transferase T1; HADHA, enoyl-CoA hydratase; NDUFA3, complex I-B9. The mean abundance of each protein is corrected to β-actin was used as a loading reference (*n* = 3). ^*^P < 0.05, ^**^P < 0.01. Abbreviations: CON = basal diet; 1.5S-OHMet = basal diet + 1.5 g/kg OHMet; 3.0S-OHMet = basal diet + 3.0 g/kg OHMet; 3.0S-Met = basal diet + 3.0 g/kg Met

A TMT-based quantitative proteomic analysis was performed to profile the expression of proteins and identify those related to placental function. A total of 5,135 proteins were quantified across all placentas (Table S2). Protein expression levels were normalized, resulting in the identification of 87 differentially expressed proteins (DEPs) (Table S3). Compared with the CON sows, 6 upregulated and 2 downregulated DEPs were found in the 1.5S-OHMet sows (Fig. 3A). Compared with the 3.0S-OHMet sows, 9 upregulated and 25 downregulated DEPs were found in the 1.5S-OHMet sows (Fig. 3B). Compared with the 3.0S-Met sows, 33 upregulated and 21 downregulated DEPs were found in the 1.5S-OHMet sows (Fig. 3C). Details of all differential proteins can be found in Table S3.

Furthermore, 24 DEPs exhibited consistent changes across the three comparative analyses. These DEPs were identified and categorized into functional groups such as amino acid metabolism (2 DEPs), cellular apoptosis (2 DEPs), complement and coagulation cascade (4 DEPs), energy metabolism (3 DEPs), immune and inflammatory responses (3 DEPs), lipid metabolism (5 DEPs), oxidative status (4 DEPs) and signal transduction mechanisms (1 DEPs) (Fig. 3D and Table 2). Compared with the CON and 3.0S-OHMet groups, the 1.5S-OHMet group showed significantly upregulated glutathione-S-transferase (GSTT1). Compared with the 3.0S-OHMet and 3.0S-Met groups, the 1.5S-OHMet group showed significantly upregulated thioredoxin (TXN) and enolase 3 (ENO3), and significantly downregulated enoyl-CoA hydratase (HADHA), apo-lipoprotein B (APOB), 2,4-dienoyl-CoA reductase 1 (DECR1), galactocerebrosidase (GALC), complex I-B9 (NDUFA3), and hemoglobin subunit zeta (HBZ) in the placenta. Compared with the 3.0S-Met group, the 1.5S-OHMet group had significantly upregulated cathepsin B (CTSB), pro-cathepsin H (CTSH), and protein disulfide-isomerase (P4HB), and significantly downregulated C3/C5 convertase (CFB), bactericidal permeability-increasing protein (BPI), histone deacetylase 3 (HADC3) and TNF-α induced protein 8 (TNFAIP8) (Fig. 3D and Table 2).

**Table 2:** Identification of differential expressed proteins in placenta

Accession	Gene name	Description	CON vs. 1.5S-OHMet		1.5S-OHMet vs. 3.0S-OHMet		1.5S-OHMet vs. 3.0S-Met		Class
			FC^1^	*P*^2^	FC^1^	*P*^2^	FC^1^	*P*^2^	
A0A5G2QYR2	*SAT2*	Thialysine N-epsilon-acetyltransferase	-	-	1.26	0.023	-	-	Amino acid metabolism
F1RLB2	*SPINK9*	Kazal-like domain-containing protein	-	-	1.52	0.017	1.36	0.058	Amino acid metabolism
A0A5S6GR81	*CTSB*	Cathepsin B	-	-	-	-	1.23	0.022	Cellular apoptosis
A0A5G2QT38	*CTSH*	Pro-cathepsin H	-	-	-	-	1.22	0.049	Cellular apoptosis
K7GPT9	*CFB*	C3/C5 convertase	-	-	0.71	0.237	0.80	0.029	Complement and coagulation cascade
F1RGX7	*HBZ*	Hemoglobin subunit zeta	-	-	0.63	0.044	0.83	0.001	Complement and coagulation cascade
A0A287A481	*PLG*	Plasminogen	-	-	0.71	0.025	0.84	0.081	Complement and coagulation cascade
A0A5G2R920	*PROS1*	Vitamin K-dependent protein S	-	-	-	-	0.81	0.025	Complement and coagulation cascade
F1SGQ5	*ADCY4*	Adenylate cyclase type 4	-	-	0.80	0.008	-	-	Energy metabolism
A0A5G2RCS9	*ENO3*	2-phospho-D-glycerate hydrolyase	-	-	1.22	0.070	1.29	0.026	Energy metabolism
F1RNI5	*NDUFA3*	Complex I-B9	-	-	0.75	0.010	0.83	0.024	Energy metabolism
A0A287BLV6	*BPI*	Bactericidal permeability-increasing protein	-	-	-	-	0.79	0.022	Immune and inflammatory responses
F2Z4Z6	*HDAC3*	Histone deacetylase 3	-	-	-	-	0.78	0.009	Immune and inflammatory responses
A0A287BL33	*TNFAIP8*	TNF alpha induced protein 8	-	-	-	-	0.83	0.004	Immune and inflammatory responses
F1SCV9	*APOB*	Vitellogenin domain-containing protein	-	-	0.80	0.017	0.78	0.017	Lipid metabolism
A0A5G2R5W7	*DECR1*	2,4-dienoyl-CoA reductase 1	-	-	-	-	0.82	0.010	Lipid metabolism
F1SDZ2	*GALC*	Galactocerebrosidase	-	-	0.82	0.073	0.79	0.003	Lipid metabolism
A0A5G2R3M6	*HADHA*	Enoyl-CoA hydratase	-	-	0.88	0.007	0.84	0.026	Lipid metabolism
I3LJL9	*MTMR8*	Phosphatidylinositol-3-phosphate phosphatase	-	-	0.82	0.003	0.80	0.051	Lipid metabolism
F1RL37	*GSTT1*	Glutathione transferase	0.82	0.043	1.21	0.032	-	-	Oxidative status
G9F6X8	*P4HB*	Protein disulfide-isomerase	-	-	-	-	1.38	0.014	Oxidative status
A0A5G2QU43	*SDR16C5*	Short chain dehydrogenase/reductase family 16C member 5	-	-	-	-	0.75	0.031	Oxidative status
A0A5G2Q9S8	*TXN*	Thioredoxin	-	-	1.27	0.045	1.47	0.035	Oxidative status
F1RV28	*MAP2K6 *	Mitogen-activated protein kinase 6	-	-	1.20	0.071	1.24	0.042	Signal transduction mechanisms

^1^FC (fold change) values were obtained from the mean peak area of the former sow/mean peak area of the latter sow. If the FC value is greater than 1, it indicates that the protein level is higher in the former group than in the latter group

^2^*P*-values were calculated from the student’s *t*-test with a threshold of 0.05

Four DEPs were chosen to validate the proteomic results by western blotting (Fig. 3E–H). TXN was downregulated and NUDFA3 was upregulated in both 3.0S-OHMet and 3.0S-Met groups compared with the 1.5S-OHMet group. Moreover, GSTT1 was upregulated in the 1.5S-OHMet group compared with both the CON and 3.0S-OHMet groups. Additionally, HADHA was upregulated in the 3.0S-OHMet and 3.0S-Met groups compared with the 1.5S-OHMet group.

### GO and KEGG enrichment analyses of DEPs

**Fig. 4:**
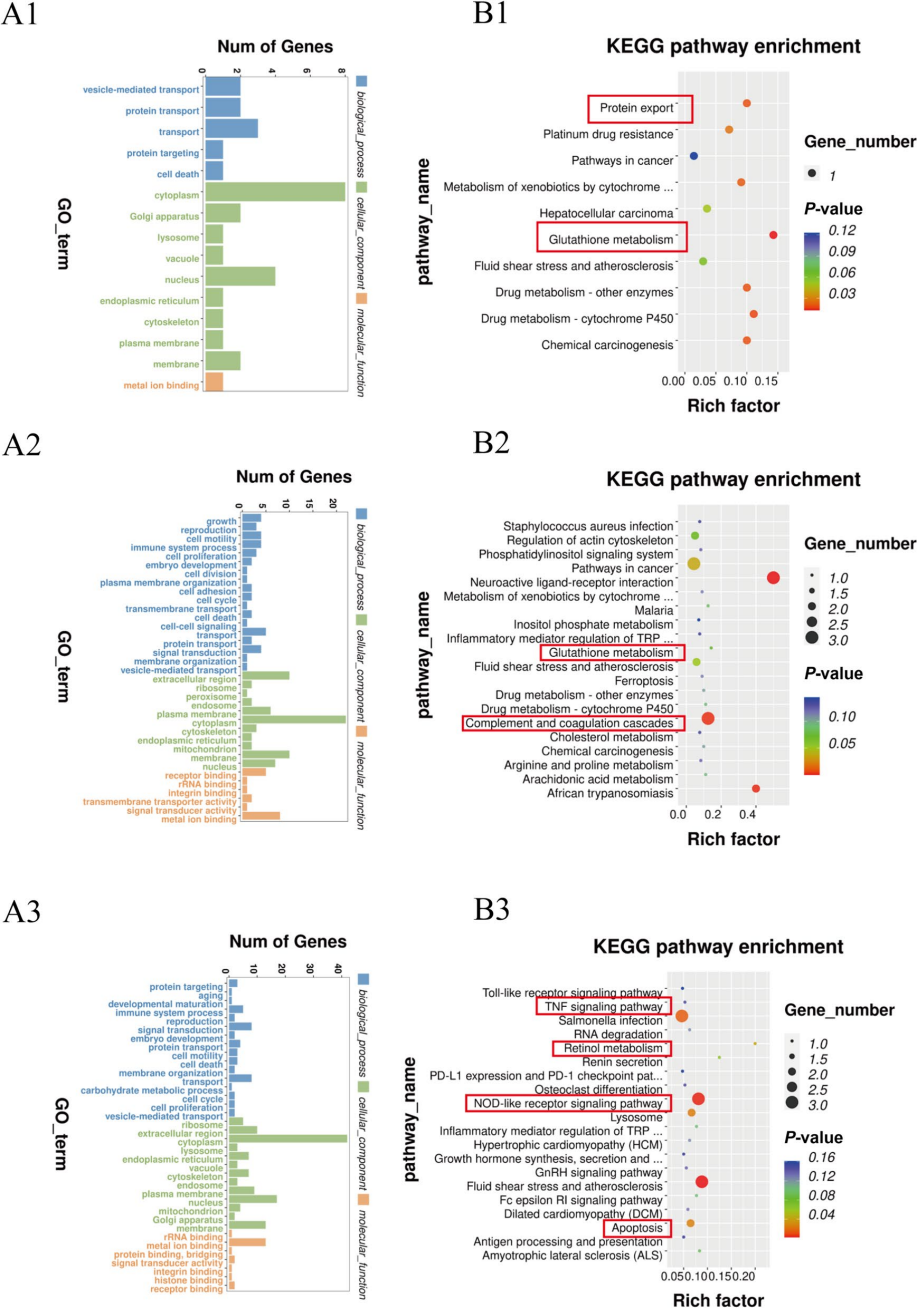
Functional characterization of differentially expressed proteins. **A1**–**A3** GO enrichment analysis of differentially expressed proteins, based on the categories of biological process, cellular component, and molecular function, between CON and 1.5S-OHMet, 1.5S-OHMet and 3.0S-OHMet, 1.5S-OHMet and 3.0S-Met, respectively. **B1**–**B3** The KEGG pathway of differential proteins between CON and 1.5S-OHMet, 1.5S-OHMet and 3.0S-OHMet, 1.5S-OHMet and 3.0S-Met, respectively. Abbreviations: CON = basal diet; 1.5S-OHMet = basal diet + 1.5 g/kg OHMet; 3.0S-OHMet = basal diet + 3.0 g/kg OHMet; 3.0S-Met = basal diet + 3.0 g/kg Met

GO enrichment and KEGG pathway analysis were performed to explore the molecular mechanisms underlying the differential expression of proteins involved in placental function (Fig. 4 and Table S4, S5). In the comparison between 1.5S-OHMet and CON placentas, the most enriched GO pathways were “transport”, “cytoplasm” and “nucleus” (Fig. 4A1), and the enriched KEGG pathways included “glutathione metabolism” and “protein export” (Fig. 4B1). For the placental DEPs in 1.5S-OHMet compared to 3.0S-OHMet placentas, the most enriched GO pathways were “extra-celluar region”, “cytoplasm” and “membrane” (Fig. 4A2), while the enriched KEGG pathways included “complement and coagulation cascades” and “glutathione metabolism” (Fig. 4B2). For the placental DEPs in 1.5S-OHMet compared to 3.0S-Met placentas, the most enriched GO pathways were “cytoplasm”, “nucleus” and “metal ion binding” (Fig. 4A3); while the enriched pathways of KEGG included “NOD-like receptor signaling pathway”, “retinol metabolism” and “apoptosis” (Fig. 4B3).

### Association between the differential proteins and placental traits

**Fig. 5:**
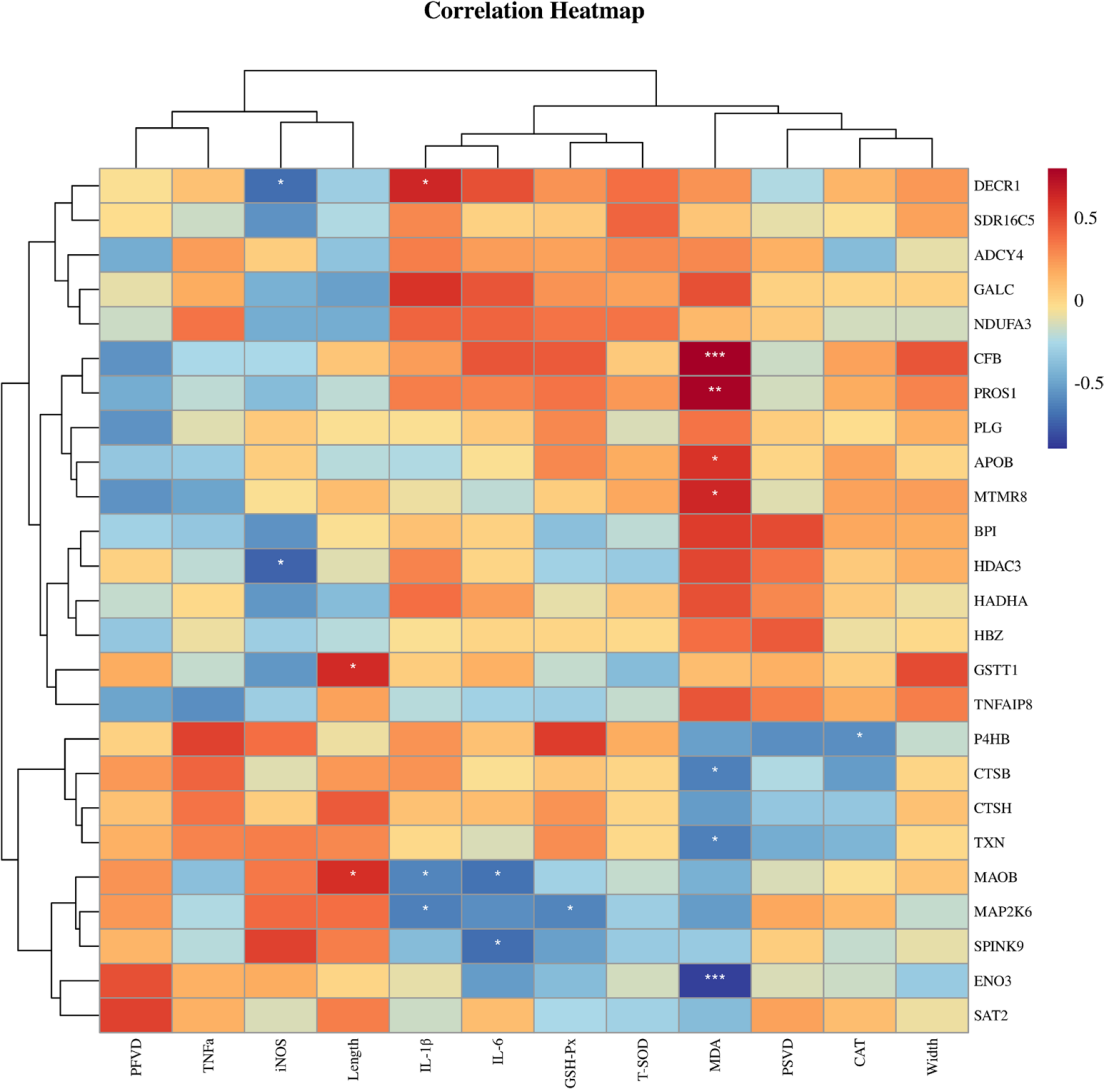
The correlation of placental differential proteins and placental indexes. PSVD, density vascular of placental stroma; PFVD, capillaries per unit area of the fold; Width, fold width; Length, placental fold length; MDA, malondialdehyde; GSH-Px, activity of glutathione peroxidase; SOD, superoxide dismutase; CAT, catalase; iNOS, inducible nitric oxide synthase; TNFα, tumor necrosis factor α; IL-6, interleukin 6; IL-1β, interleukin 1β. ^*^P < 0.05, ^**^P < 0.01, ^***^P < 0.001

As shown in Fig. 5, antioxidative indexes determined that the placental MDA levels exhibited negative correlations with the changes in ENO3, TXN and CTSB, and positive correlations with the changes in CFB, PROS1 APOB and MTMR8. Additionally, inflammation indexes revealed that the IL-1β levels were negatively correlated with changes in amine oxidase (MAOB) and mitogen-activated protein kinase 6 (MAP2K6), and positively correlated with changes in DECR1. Moreover, the IL-6 levels were negatively correlated with changes in MAOB and Kazal-type protein 1 (SPINK).

### Association between the differential proteins and maternal methionine metabolites

**Fig. 6:**
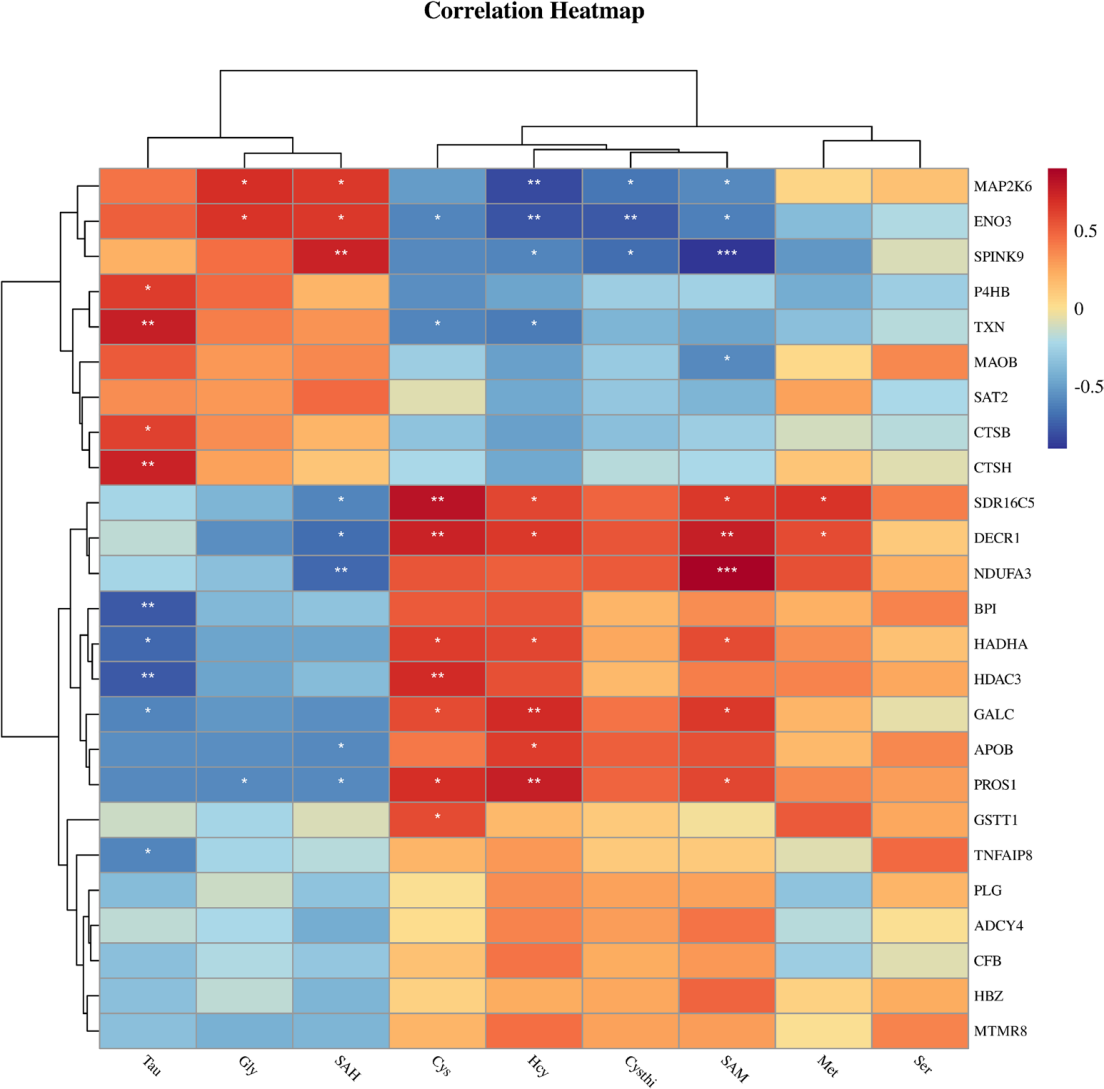
The correlation of placental differential proteins and maternal methionine metabolites. Met, methionine; SAM, *S*-adenosyl-methionine; SAH, *S*-adenosyl-homocysteine; Hcy, homocysteine; Cysthi, cystathionine; Tau, taurine; Ser, serine; Gly, glycine. ^*^P < 0.05, ^**^P < 0.01, ^***^P < 0.001

To investigate the relationship between Met metabolites and DEPs, heat maps were generated by Pearson correlation analysis (Fig. 6). The Met metabolites data were obtained from our previous study [17]. Hcy levels were negatively correlated with changes in TXN and ENO3, and positively correlated with changes in HADHA, APOB, DECR1, and GALC. Cys levels were positively correlated with changes in GSTT1. In addition, SAM levels were positively correlated with changes in NDUFA3, and negatively correlated with changes in SPINK9. Tau levels were positively correlated with changes in TXN and P4HB, and negatively correlated with changes in HADHA, HDAC3, BPI, TNFAIP8, and GALC3.

### Increased consumption of methionine as OHMet improved angiogenesis and antioxidative capacity in vitro

**Fig. 7:**
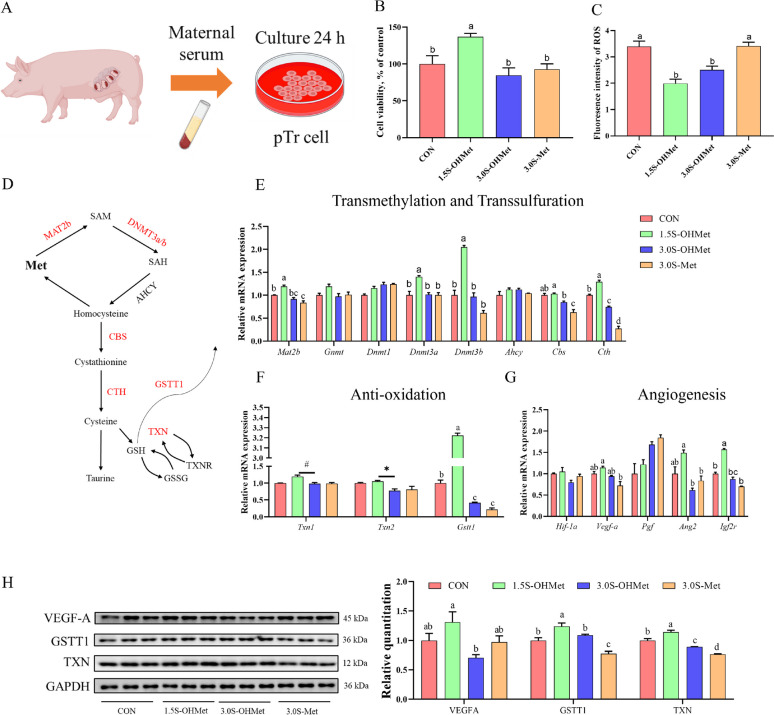
Increased consumption of methionine as OHMet improved angiogenesis and antioxidative capacity in pTr cells. **A** The trophoblast cells were treated for 24 h with 20% serum from each group (*n* = 3). **B** Cell viability. **C** Reactive oxidative species level. **D** Schematic of the Met metabolism and list of genes directly involved and associated with this pathway. **E** The gene expression of transmethylation and trans-sulphuration. **F** The gene expression of anti-oxidation. **G** The gene expression of angiogenesis. **E**–**G** Duplicated independent experiments. **H** The protein expression of pTr, GAPDH as a loading control. TXN, thioredoxin; GSTT1, glutathione transferase T1; VEGF-A, vascular endothelial growth factor A. Significant difference among four treatments is marked with different superscripts (*P* < 0.05). Abbreviations: CON = basal diet; 1.5S-OHMet = basal diet + 1.5 g/kg OHMet; 3.0S-OHMet = basal diet + 3.0 g/kg OHMet; 3.0S-Met = basal diet + 3.0 g/kg Met

To confirm the improved antioxidative capacity and angiogenesis from maternal supplementation with 1.5S-OHMet, pTr cells were cultured using maternal serum from each treatment (Fig. 7A). Serum from the 1.5S-OHMet group significantly increased pTr cell viability and decreased ROS levels compared with other groups (Fig. 7B and C). Furthermore, serum from the 1.5S-OHMet group upregulated the gene expression of *Mat2b*, *Dnmta*, *Dnmt3b*, and *Cth* compared with other groups, and also upregulated the gene expression of *Cbs*, *Gstt1*, *Ang2*, and *Igf2r* compared with the 3.0S-OHMet and 3.0S-Met groups (Fig. 7E–G). Serum from the 3.0S-OHMet group upregulated the gene expression of *Cth* and* Cbs* compared with the 3.0S-Met group (Fig. 7E). Serum from the 1.5S-OHMet group upregulated the gene expression of *Vegf-a* compared with the 3.0S-Met group (Fig. 7G). As shown in Fig. 7H, serum from the 1.5S-OHMet group upregulated the protein expression of GSTT1 and TXN compared with other groups, and upregulated the protein expression of VEGF-A compared with the 3.0S-OHMet group.

### Hcy induced anti-proliferative effects and reduced antioxidant capacity of pTr cells

**Fig. 8:**
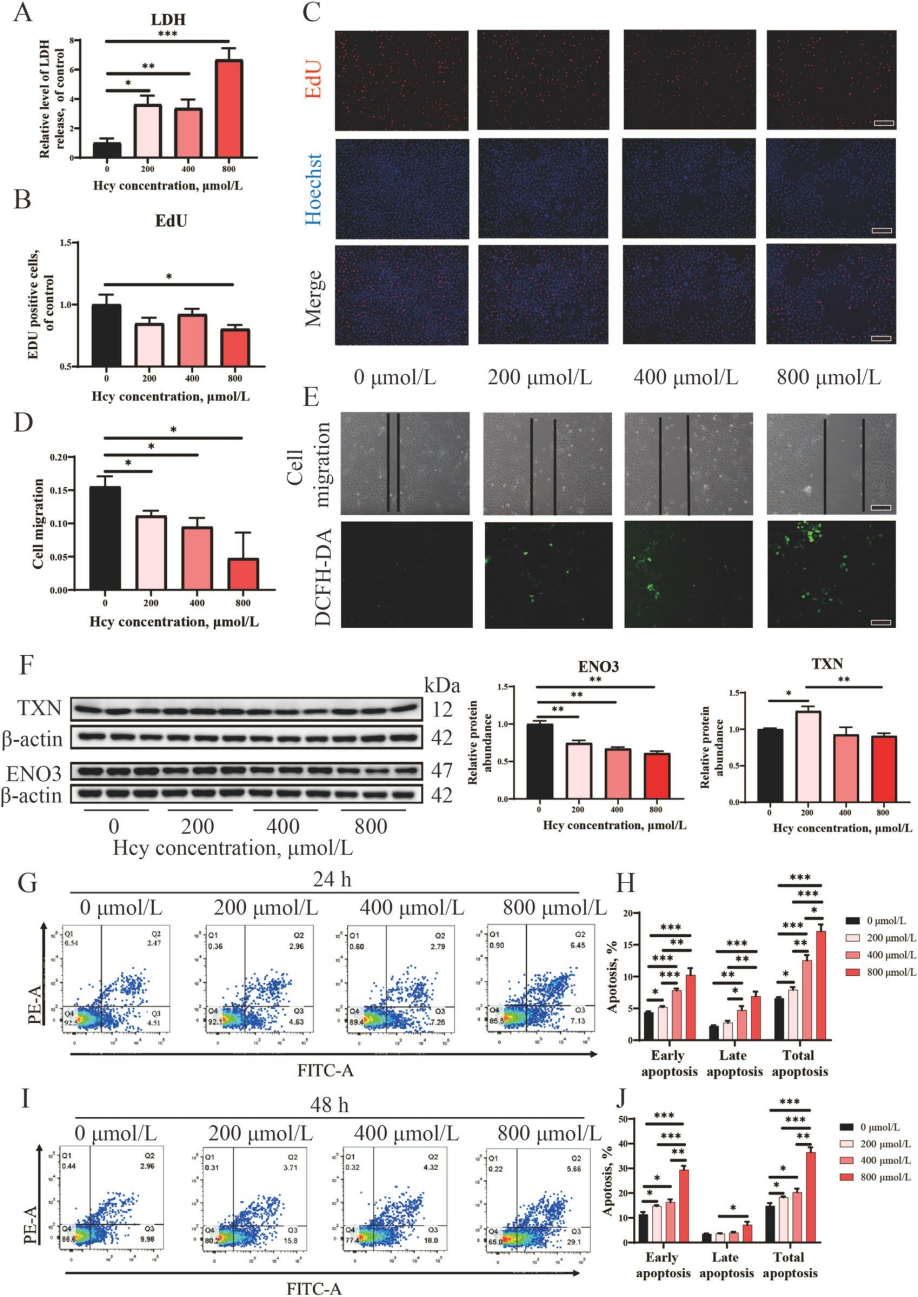
Anti-proliferative and inhibited antioxidant capacity induced by homocysteine in pTr cells (*n* = 3). **A** Detection of LDH activity in pTr cells. **B** EdU-positive cells were measured using ImageJ software. **C** Cell proliferation was determined by the EdU assay, and images were taken under a fluorescence microscope (magnification 400 × , bar = 100 μm). **D** Cell migration was measured using ImageJ software. **E** The cell migration distance (bar = 500 μm) and images of ROS were taken using a DCFH-DA probe (magnification 400 × , bar = 500 μm). **F** Representative western blot results for protein levels of enolase 3 (ENO3) and thioredoxin (TXN), β-actin was used as a loading reference. **G**–**J** Detection of apoptosis in pTr cells. Annexin V and propidium iodide (PI) fluorescence was quantified using flow cytometry. ^*^P < 0.05, ^**^P < 0.01, ^**^^*^P < 0.001

To further investigate the effect of Hcy on the proliferation and antioxidant capacity of pTr cells, various concentrations of Hcy were administered to the pTr cells. Compared to the 0 μmol/L Hcy group, the release of LDH was observed in pTr cells at Hcy concentrations of 200, 400, and 800 μmol/L (Fig. 8A). Moreover, treatment of pTr cells with 800 μmol/L Hcy significantly reduced cell proliferation (Fig. 8B and C). Additionally, cells treated with 200, 400, and 800 μmol/L Hcy had impaired cell migration and increased ROS levels (Fig. 8D and E). The protein expression of ENO3 was significantly inhibited when pTr cells were treated with Hcy concentrations of 200, 400, and 800 μmol/L (Fig. 8F). The protein expression of TXN was significantly inhibited at 800 μmol/L Hcy compared with the 200 μmol/L Hcy group (Fig. 8F). Finally, Hcy was found to increase the percentage of apoptosis in pTr cells at 24 and 48 h (Fig. 8G–J).

## Discussion

The placenta is responsible for nutritional support and exchange between the mother and fetus, directly affecting fetal growth and development [23]. Previous studies have shown that Met metabolism during late pregnancy noticeably influences both placental vessel density and antioxidant capacity [6]. Growing evidence suggests that Met supplementation can improve the reproductive performance of sows by promoting maternal metabolism and enhancing placental function [8, 10]. However, the mechanisms by which Met nutrition improves placental development are unclear. Thus, this study aimed to explore how various levels and sources of Met regulate placental function.

### Consumption of 1.5S-OHMet improved placental angiogenesis by enhancing antioxidant capacity

In the current study, sows supplemented with 1.5S-OHMet exhibited the highest number of capillaries per unit area of placental fold, aligning with previous findings that maternal dietary Met supplementation during late gestation enhances placental angiogenesis [6]. Furthermore, serum from the 1.5S-OHMet group upregulated the mRNA expression of *Ang2* and *Vegf-α* in pTr cells and increased the protein expression of VEGF-A. This upregulation of VEGF-A facilitated angiogenesis by its interaction with VEGFR [2]. Notably, the KEGG enrichment analysis of DEPs identified pathways linked to antioxidant capacity. Sows on 1.5S-OHMet diets exhibited upregulated protein expression of GSTT1 and TXN in both placenta and pTr cells, indicating an enhanced antioxidant capacity. This finding is consistent with our earlier work, which showed that increased maternal intake of OHMet improved the antioxidant capacity of the neonatal intestine [24]. Overall, these results suggest that maternal supplementation with 1.5S-OHMet improves placental angiogenesis and oxidative status.

### Excessive Met consumption reduced placental antioxidant capacity via accumulating Hcy

Our previous study indicated a significant rise in Hcy levels as pregnancy progressed, with excessive Met consumption exacerbating Hcy accumulation [17]. Given that Hcy is known to impact early extra-embryonic angiogenesis by reducing antioxidant capacity [25], we suggest that the placental oxidative stress in the 3.0S-Met group may be attributed to elevated maternal Hcy levels. Previous studies have shown that high Hcy concentration can lead to the generation of ROS, downregulation of TXN, increased MDA levels, inhibition of iNOS activity, and ultimately contribute to oxidative stress and cell apoptosis [12, 26, 27]. Consistent with our findings, the 3.0S-Met group showed increased placental MDA levels and apoptosis-related CTSB protein expression, along with decreased TXN protein expression, compared to the 1.5S-OHMet group. Furthermore, we observed a significant negative correlation between maternal Hcy levels and placental TXN expression, corroborating earlier studies [28]. Further studies confirmed that elevated Hcy concentrations inhibit TXN expression and increase ROS levels and the rate of apoptosis in pTr cells. Furthermore, we observed a negative correlation between placental MDA levels and placental TXN expression. MDA, a byproduct of lipid peroxidation, serves as an indicator of oxidative stress [29]. These results suggest that elevated Hcy levels lead to increased MDA levels and decreased TXN expression in the placenta, thus reducing its antioxidant capacity.

Further analysis revealed elevated expression of placental APOB, DECR1, and HADHA proteins in sows fed diets containing 3.0S-OHMet or 3.0S-Met compared to those fed 1.5S-OHMet. DECR1 and HADHA are recognized for their roles in the beta-oxidation pathway of fatty acids, whereas APOB is implicated in lipid transport [30, 31]. We propose that placentas from sows on 3.0S-OHMet and 3.0S-Met diets experience greater lipid catabolism compared to those on 1.5S-OHMet diets. This suggests that the 3.0S-Met diets are associated with lipid peroxidation. Moreover, the increased MDA levels in placentas from sows on 3.0S-Met diets indicate lipid peroxidation. Similar findings were reported by Zhou et al., which indicated that ectopic lipid accumulation can lead to lipotoxicity in the placenta [32].

Studies have shown that maternal obesity can lead to placental lipotoxicity, which induces inflammation and oxidative stress [33]. Consistent with the current study, sows fed the 1.5S-OHMet diet exhibited downregulated placental TNFAIP8 protein expression and upregulated P4HB expression compared to those fed the 3.0S-Met diet. TNFAIP8 binds to fatty acids, regulates the expression of enzymes involved in lipid and fatty acid metabolism, and modulates autophagy and cellular steatosis [34]. Furthermore, TXN and protein disulfide isomerase (P4HB), components of the thioredoxin system, play essential roles in modulating oxidative stress [35]. These results suggested that maternal consumption of the 1.5S-OHMet diet reduced the oxidation of lipid metabolites by enhancing antioxidant capacity, whereas 3.0S-Met supplementation may disrupt placental lipid metabolism.

Furthermore, maternal 3.0S-OHMet or 3.0S-Met diet consumption increased placental IL-1β levels. Sows fed a 3.0S-Met diet had upregulated placental HDAC3 expression compared with those fed CON or 1.5S-OHMet diet. Oxidative stress activates NF-κB via ROS production, resulting in the production of IL-1β and IL-6, and subsequent inflammation [36, 37]. Additionally, HDAC3 promotes acute inflammation induced by lipopolysaccharide by enhancing NLRP3-dependent caspase-1 activation [38]. In our previous study, sows fed 3.0S-Met had decreased serum butyric acid levels compared to those fed 1.5S-OHMet [17]. Butyric acid is reported to mitigate tissue inflammation by inhibiting HDAC3 expression and prevent oxidative stress by reducing ROS production [39]. These results suggest that the reduced butyric acid level may be one cause of placental inflammation. Moreover, placentas from dietary supplementation with 1.5S-OHMet exhibited lower PLG expression and lower CFB expression compared to those from the 3.0S-OHMet or 3.0S-Met groups. Upregulation of CFB promotes the activation of the C3 convertase enzyme [40]. PLG binds to C5 and C3 resulting in increased levels of C4b-binding protein, suggesting potential interactions during acute inflammation [41]. These results indicate that 1.5S-OHMet supplementation improves lipid metabolism and reduces inflammation, whereas excessive Met supplementation may induce placental inflammation.

### Excessive Met consumption affects placental energy metabolism via accumulating Hcy

Maternal nutrition influences placental function, modulating energy metabolism [42]. Ensuring adequate energy supplementation for sows during farrowing is essential for preventing fetal mortality [43]. Analysis of differential protein expression in the placenta revealed that sows fed diets containing 3.0S-Met or 3.0S-OHMet had decreased levels of ENO3 protein and increased levels of NDUFA3 protein compared to those fed 1.5S-OHMet. Notably, the ENO3 protein participated in the pathway synthesizing pyruvate from D-glyceraldehyde 3-phosphate [44]. Downregulation of the ENO3 protein in placentas from sows fed 3.0S-Met and 3.0S-OHMet diets suggests a potential reduction in phosphoenolpyruvate synthesis. Furthermore, NDUFA3, a component of Complex I, plays a role in generating ROS during oxidative phosphorylation [45]. In our previous study, sows fed 3.0S-Met and 3.0S-OHMet diets had higher succinate levels than those fed 1.5S-OHMet diet [17]. Therefore, we hypothesize that an inadequate energy supply may have led to the compensatory upregulation of NDUFA3 in placentas of sows fed the 3.0S-Met and 3.0S-OHMet diets. Additional experiments demonstrated that increased Hcy levels significantly suppressed ENO3 expression in pTr cells, confirming that excessive maternal Met supplementation can disrupt energy metabolism and impair placental function.

### OHMet resulted in lower accumulation of Hcy compared to Met

During late gestation, significant changes in maternal Met metabolites occurred [10], impacting maternal glycolipid and amino acid metabolism [17]. To further explore the alterations in placental Met metabolism induced by difference in Met nutrition, the gene expression of the transmethylation and trans-sulphuration pathways in pTr cells was measured. Intriguingly, serum from the 1.5S-OHMet group increased the mRNA levels of *Mat2b*, *Dnmt3a*, and *Dnmt3b* in vitro, indicating that 1.5S-OHMet promote the transmethylation pathway. Expression of MAT2B was increased in the OHMet group, which facilitates SAM biosynthesis [46]. The increased expression of DNMT3A and DNMT3B may suggest that OHMet improves placental methylation levels [47]. Additionally, a previous study suggested that OHMet could promote the transsulfuration of Met [48]. In line with our findings, the expression of *Cbs* and *Cth* genes in the 3.0S-OHMet group was significantly higher than those in the 3.0S-Met group. These findings suggest that OHMet results in lower Hcy accumulation than crystalline Met through the trans-sulphuration pathway [49].

Our research had limitations. Firstly, it was unclear whether the improvement in placental function was achieved through OHMet itself or its metabolites. Secondly, the optimal dosage of OHMet was not determined and further research is warranted to determine the dosage for improving the reproductive performance of gestation sows.

## Conclusion

Maternal supplementation with 1.5S-OHMet improved placental development by promoting angiogenesis and enhancing antioxidative capacity. In contrast, consumption of 3.0S-OHMet or 3.0S-Met diets resulted in increased placental inflammatory cytokine levels, disturbed lipid metabolism, and altered oxidative status, attributed to increased Hcy levels. These findings underscore the importance of optimizing maternal Met intake levels and sources during late pregnancy to enhance placental function.

## Supplementary Information

Additional file 1: Fig. S1. The protein detection for placental samples.

Additional file 2: Table S1. Primers used for quantitative real-time PCR (qPCR). Table S2. A total of 5,136 proteins were quantified from all the placentas. Table S3. Differentially abundant proteins (DAPs) identified in the placentas of sows from the CON vs. 1.5S-OHMet, 1.5S-OHMet vs. 3.0S-OHMet and 1.5S-OHMet vs. 3.0S-Met groups. Table S4. Significantly enriched KEGG pathway of the differentially abundant proteins (DAPs). Table S5. Significantly enriched GO Slim terms of the differentially abundant proteins (DAPs).

## Data Availability

The datasets analyzed in the current study are available from the corresponding author on reasonable request.

## Abbreviations

APOB: Apo-lipoprotein B
BPI: Bactericidal permeability-increasing protein
CAT: Catalase
CTSB: Cathepsin B
CTSH: Pro-cathepsin H
CFB: C3/C5 convertase
DECR1: 2,4-Dienoyl-CoA reductase 1
ENO3: Enolase 3
FW: Fold width
GALC: Galactocerebrosidase
GSH-Px: Glutathione peroxidase
GSH: Reduced glutathione
GSSG: Oxidative glutathione
Hcy: Homocysteine
HADHA: Enoyl-CoA hydratase
HBZ: Hemoglobin subunit zeta
HADC3: Histone deacetylase 3
iNOS: Inducible nitric oxide synthase
IL-1β: Interleukin 1β
IL-6: Interleukin 6
MDA: Malondialdehyde
NDUFA3: Complex I-B9
SOD: Superoxide dismutase
SAM: *S*-adenosyl-methionine
SAH: *S*-adenosyl-homocysteine
PSVD: Density vascular of placental stroma
PFVD: Capillaries per unit area of the fold
P4HB: Protein disulfide-isomerase
PFL: Placental fold length
TXN: Thioredoxin
TNFAIP8: TNF-α induced protein 8
VEGF-A: Vascular endothelial growth factor A
