# Supplementary material for: Increased maternal consumption of methionine as its hydroxyl analog improves placental angiogenesis and antioxidative capacity in sows
Source: J Anim Sci Biotechnol. 2025 Mar 9;16:39. doi: 10.1186/s40104-025-01159-z (PMC11890719; doi:10.1186/s40104-025-01159-z)
Supplement: Supplementary file 1 — Additional file 1: Fig. S1. The protein detection for placental samples. [file 40104_2025_1159_MOESM1_ESM.docx]

**Increased maternal consumption of methionine as its hydroxyl analog** **improves placental angiogenesis and antioxidative capacity in sows**

Rui Zhou^1,2^, Shanshan Lai^1^, Peiqiang Yuan^1^, Li Zhe^1^, Lunxiang Yang^1^, Yves Mercier^3^, Liang Hu^2^, Xiaoling Zhang^1^, Lun Hua^1^, Yong Zhuo^1^, Shengyu Xu^1^, Yan Lin^1^, Bin Feng^1^, Lianqiang Che^1^, De Wu^1^, Zhengfeng Fang^1,2,*^

^1^Key Laboratory for Animal Disease-Resistance Nutrition of China Ministry of Education, Animal Nutrition Institute, Sichuan Agricultural University, 211 Huimin Road, Wenjiang District, Chengdu, 611130, People’s Republic of China;

^2^Key Laboratory of Agricultural Product Processing and Nutrition Health, Ministry of Agriculture and Rural Affairs, College of Food Science, Sichuan Agricultural University, Ya’an 625014, People’s Republic of China;

^3^Adisseo France S.A.S., CERN, Commentry, France

*Correspondence: ZFang@sicau.edu.cn


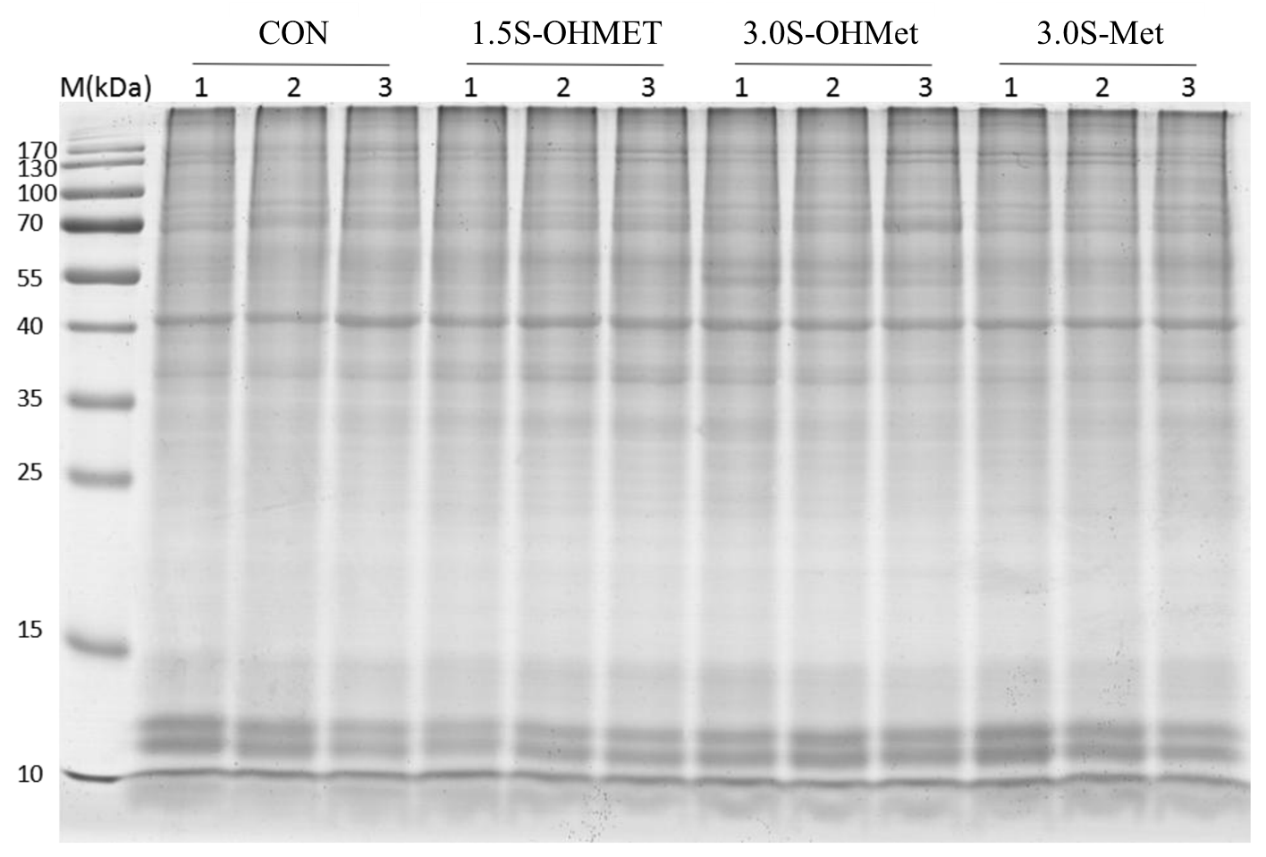


Fig. S1. Protein detection of placental samples.
